# The Effect of Emulsion Intensity on Selected Sensory and Instrumental Texture Properties of Full-Fat Mayonnaise

**DOI:** 10.3390/foods7010009

**Published:** 2018-01-17

**Authors:** Viktoria Olsson, Andreas Håkansson, Jeanette Purhagen, Karin Wendin

**Affiliations:** 1 Research Environment MEAL, Faculty of Natural Science, Kristianstad University, SE-291 88 Kristianstad, Sweden; andreas.hakansson@hkr.se (A.H.); karin.wendin@hkr.se (K.W.); 2 Perten Instruments AB, SE-254 66 Helsingborg, Sweden; jpurhagen@perten.com; 3 Department of Food Technology, Engineering and Nutrition, Lund University, SE-221 00 Lund, Sweden; 4 Department of Food Science, University of Copenhagen, DK-1958 Frederiksberg, Denmark

**Keywords:** mayonnaise, emulsification, sensory evaluation, texture, processing

## Abstract

Varying processing conditions can strongly affect the microstructure of mayonnaise, opening up new applications for the creation of products tailored to meet different consumer preferences. The aim of the study was to evaluate the effect of emulsification intensity on sensory and instrumental characteristics of full-fat mayonnaise. Mayonnaise, based on a standard recipe, was processed at low and high emulsification intensities, with selected sensory and instrumental properties then evaluated using an analytical panel and a back extrusion method. The evaluation also included a commercial reference mayonnaise. The overall effects of a higher emulsification intensity on the sensory and instrumental characteristics of full-fat mayonnaise were limited. However, texture was affected, with a more intense emulsification resulting in a firmer mayonnaise according to both back extrusion data and the analytical sensory panel. Appearance, taste and flavor attributes were not affected by processing.

## 1. Introduction

Mayonnaise is an oil-in-water emulsion stabilized by egg yolk and has been produced commercially for more than one hundred years [1]. Traditional mayonnaise is produced in a batch process by slowly adding the oil to the water phase under vigorous mixing, thereby creating an emulsion [2]. Industrially, mixing is achieved using high-intensity rotor-stator mixers, also referred to as high-shear mixers [3]. Although the taste and texture of mayonnaise is appreciated by many consumers, local markets often value different sensory properties. Therefore, as it is known that production techniques such as mixing/homogenization may have a considerable effect on the final product structure [1,4], better knowledge of how processing conditions affect the sensory and instrumental properties of the emulsion could help cater for such varying consumer preferences.

Due to a high oil content, mayonnaise exhibits a semisolid and viscoelastic behavior that influences its particular rheological properties, which in turn contribute to the perceived texture and flavor of the product [5]. In this context, texture is defined as the sensory perception of the structure of a food [6]. According to van Aken et al. [7], the rheological properties of a food product are very important for the perception of a creamy mouthfeel, although other authors have stressed that a variety of aspects may also play a role. For example, the oil droplet size is another parameter of interest due to its ability to influence product appearance, texture, and flavor profile [8].

One way in which the texture of mayonnaise is perceived by the consumer is through its processing and breakdown in the mouth (intra orally) before it is swallowed. In fact, most sensations associated with food texture occur only when the food is manipulated, deformed, or moved across the receptors in the mouth [4]. Through texture analysis, it is possible to choose a compression technique similar to that performed by the mouth, and then measure the behavior of the food using this technique. Such tests are valuable since they can confirm various textural properties, including the creaminess of mayonnaise.

Texture is also perceived outside the mouth (extra orally). Before the food item enters the mouth, visual cues related to the item’s appearance provide information regarding its texture, while additional information can also be obtained by handling the food, e.g., by stirring, spooning, and cutting [4].

The emulsification taking place when mayonnaise is formed in rotor-stator mixers is relatively well understood, and proceeds via hydrodynamic interactions between the dispersed phase and the fluid in the rotor-stator region. Experiments suggest that the dispersed phase is predominantly broken up by turbulent viscous stresses [9]. The diameter of the emulsion drops, *U*, is produced in the rotor-stator mixer scales with the rotor tip-speed, determined by

(1) U=πND

and decreases according to the power-law function [9,10]. In Equation (1), *N* is the rotor speed and *D* is the rotor diameter. Drop size also decreases with processing time, and scales with the average number of passages, *p*, through the rotor-stator region [9], which is written as

(2) $$p=t\frac{Q}{V}$$

where *t* is processing time, *V* is the fluid volume, and *Q* is the flow through the stator screen of the mixer [11,12], expressed as follows:

(3) $$Q=N_{Q}ND^{3}$$

where *N_Q_* is a mixer-specific design constant. However, the dynamics of the process is very slow and the droplet size continues to decrease after the emulsion has been processed for more than the equivalent of an average of 100 passages through the rotor-stator region [9].

The effect of processing conditions on the sensory response of mayonnaise is not as well understood as the effect on emulsion drop diameters. Furthermore, studies in which the rheological properties of mayonnaise have been related to perceived texture have predominantly focused on low-fat mayonnaises with oil concentrations ranging from 15–30% [13], thus creating a knowledge gap with regard to how full-fat (~75–80%) mayonnaises are affected. 

It has been shown that fat content has a significant effect on perceived thickness and fattiness, with a higher fat content yielding a higher perception of both qualities. However, increased emulsification intensity, which produces smaller droplets, has the opposite effect and has also been shown to affect the perceived sweetness and whiteness of mayonnaise with added aromas [14]. Taste, flavor and textural attributes are also of interest in mayonnaise without added aromas.

The aim of this study was to evaluate the effect of emulsification intensity on the sensory and instrumental characteristics of full-fat mayonnaise.

## 2. Materials and Methods

### 2.1. Mayonnaise Ingredients and Processing

The ingredients in the experimental mayonnaise are presented in Table 1 and were assembled according to the following protocol: All ingredients, with the exception of the rapeseed oil and vinegar, were weighed into a 500 mL dispersing vessel (Kinematica, Luzern, Switzerland) and allowed to rest for adjustment to room temperature. The dispersing aggregate PT-DA 20/2 EC-E192 (Kinematica, Luzern, Switzerland) of the rotor-stator mixer (Kinematica Polytron, PT 2500 E, Luzern, Switzerland) was adjusted in the vessel and the mixture processed for 30 s at 6000 rpm. The rapeseed oil was then added, initially dropwise, and the mixture processed at 6000 rpm until all oil was emulsified. Following this stage, the vinegar was added and the mayonnaise mixed for an additional 30 s. Thereafter, one batch (High emulsification intensity) was processed at 9000 rpm (corresponding to a rotor tip-speed of 7.1 m/s) for 6 min, and a second batch (Low emulsification intensity) at 6000 rpm (rotor tip-speed 4.7 m/s) for 9.2 min. These processing times were chosen to achieve the same number of average rotor-stator passages for each rotor speed, i.e., equal *p* for different *N* (see Equations (1)–(3)). This procedure was repeated 3 times per processing condition for both batches (High and Low) and pooled in order to obtain enough mayonnaise for sensory and instrumental texture analysis. A commercial mayonnaise (Äkta Majonnäs, Findus), produced in Sweden, was also included as reference; this mayonnaise contained 81% (*w*/*v*) rapeseed oil and 4.6% (*w*/*v*) egg yolk (Table 2). The rationale for including a commercial reference was dual-fold; not only did it provide an indication of the sensory characteristics of the experimental mayonnaise in comparison to those of a commercial mayonnaise sold on the Swedish retail market, but it also served as a control when comparing the results of the present panel with those of other analytical panels.

### 2.2. Sensory Evaluation

Sensory evaluation and training were carried out over a period of three days by an external panel (Kristianstad University, Kristianstad, Sweden) of ten assessors, who were selected and trained according to the following guidelines: ISO 3972, ISO8586-1, and ISO8586-2. The sensory laboratory was designed according to ISO 8589 and sensory analysis performed using sensory descriptive analysis [15]. Across two training sessions lasting approximately 2 h each, the panel developed descriptions of the perceived sensory attributes of the products, generating a set of attributes and developing a consensus regarding the evaluation of each attribute (Table 3). Reference materials were used in training for selected attributes such as yellow color, acid taste, and egg flavor.

Product evaluations were performed individually, in isolated booths. Samples (20 g) of mayonnaise were served on coded, disposable plastic dishes and handled using a plastic spoon. The serving temperature was controlled by leaving the samples at room temperature for 10 min prior to serving. Panelists were instructed to rinse their mouths with still or carbonated water after each sample, and were also provided with fresh cucumber, apple, and soft white bread for further palate cleansing. Samples were coded with three-digit codes and served in a randomized order. The panelists then evaluated the perceived intensities by hand on a continuous 100 mm line-scale labeled “low intensity” at 10 mm and “high intensity” at 90 mm. During one evaluation session, lasting 60 min, the panelists evaluated duplicates of each product, with the intensity ratings then translated into numbers. 

### 2.3. Instrumental Texture Analysis

Texture measurements were performed in duplicate, via a back extrusion method using a TVT-300XP analyzer (Perten Instruments AB, Stockholm, Sweden) equipped with a 7 kg load cell and a back extrusion set consisting of a sample container (50 mm diameter) and a compression plate (40 mm diameter). The sampling distance was 20 mm, the test speed 1 mm/s, and the retraction speed 5 mm/s. Texture properties (Table 4) were measured using the TexCalc software (version 4.0.4.67).

### 2.4. Statistical Methods

From the obtained data, mean values and standard deviations were calculated both for sensory and instrumental analysis. The sensory data were further subjected to a three-way analysis of variance (ANOVA), with samples panelists and replicates as fixed effects. The instrumental data were subjected to a one-way analysis of variance (ANOVA). Significant differences (*p* < 0.05) between samples were calculated via the Bonferroni’s pairwise comparison test (Panel Check, v. 1.4.2, https://sourceforge.net/projects/sensorytool, Nofima, Norway).

Pearson correlations between instrumental and sensory data were calculated using Excel 2013 (Office for Windows), and Principal Component Analysis (PCA) performed using Panel Check (v. 1.4.2).

Panel performance was checked by calculating *p*- and MSE-values and then plotting these values in *p*-MSE-diagrams (Panel Check, v. 1.4.2).

## 3. Results

### 3.1. Sensory Evaluation

Panelist performance was found to be reliable based on the calculation of *p*-MSE-values showing low *p*- and MSE-values for all panelists. No significant effects were obtained due to replicates, with the exception of the attribute saltiness for which there was no significant difference between products.

Mixing intensity affected extra-oral texture attributes, i.e., those obtained by handling the mayonnaise through stirring and spooning (Table 5). The higher mixing intensity (rotor tip-speed 7.1 m/s) led to a significantly firmer, more viscous mayonnaise compared to the lower mixing intensity (rotor tip-speed 4.7 m/s) when handled by spoon. The results also indicated that the higher emulsification intensity produced a mayonnaise perceived as more creamy, although not to a statistically significant level.

**Figure 2:**
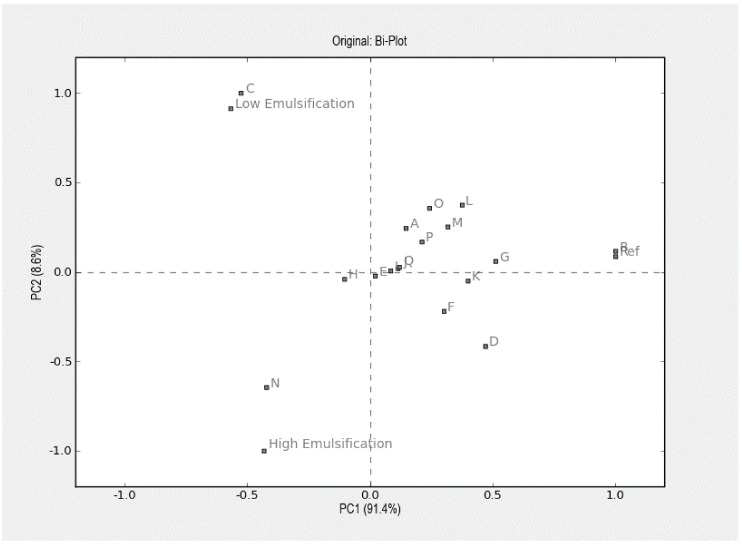
Principal component analysis (PCA) illustrating the sensory and instrumental characteristics of the experimental and commercial reference (Ref.) mayonnaises. The PCA plot shows 100% of the explained variance, meaning that the total variance is explained by two dimensions. A: Shiny; B: Yellow; C: Adhesiveness Spoon; D: Firmness; E: Fatty Mouthfeel; F: Creaminess; G: Acidity; H: Sweetness; I: Egg flavor; J: Saltiness; K: Total Flavour; L: Inst Firmness; M: Inst Compression; N: Inst Stickiness; O: Inst Adhesiveness: P: Inst Gradient 1; Q: Inst Gradient 2.

Neither appearance nor taste or flavor attributes were affected by emulsion intensity (Table 5). The commercial, reference mayonnaise stood out in the sensory evaluation as having a more pronounced yellow color and a firmer, more creamy texture when assessed both extra- and intra-orally. The panel also perceived a more intense acidic flavor and a more intense total flavor in the commercial reference mayonnaise as compared to those prepared for the study (Figure 2).

### 3.2. Instrumental Texture Analysis

**Figure 3:**
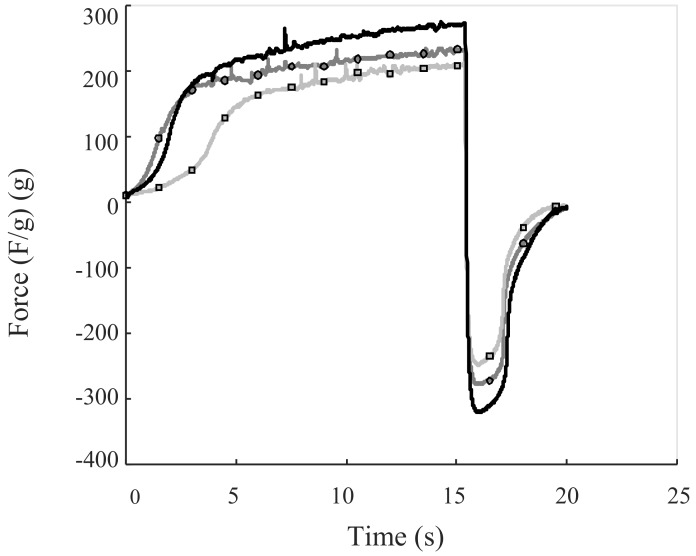
Graphs of back extrusion measurements. The black line represents the commercial reference mayonnaise, the gray line with circles the high emulsification intensity mayonnaise, and the gray line with squares the low emulsification intensity mayonnaise.

All samples exhibited the same behavior during back extrusion measurements, but to different extents, as shown in Figure 3.

The commercial reference sample was found to be the most firm, sticky and adhesive mayonnaise, followed by the mayonnaise produced with a high emulsification intensity (Table 6).

Consequently, both the sensory and instrumental data imply that a higher emulsification intensity results in a firmer full-fat mayonnaise. When correlating the instrumental data to the sensory data the correlations were generally high (*r* ≥ 0.9), primarily reflecting the pronounced difference between (i) the experimental mayonnaises (high and low emulsification intensity) and (ii) the commercial reference sample.

## 4. Discussion

A higher emulsification intensity affects the microstructure of mayonnaise by decreasing the droplet size [9,10], thus potentially affecting sensory traits such as texture [13], color [14], and flavor [8]. However, our results revealed no significant difference between the experimental samples with regard to color, taste, or flavor. As smaller particles increase light scattering, a reduced droplet size leads to a whiter mayonnaise, a phenomenon that has been illustrated in cream cheese, in which homogenization was found to lower the intensity of the yellow color [16]. In theory, flavor release decreases with increasing droplet size, as it takes longer for flavor molecules to diffuse out of a larger droplet. However, polar and non-polar flavor molecules behave differently in this respect, and the influence of droplet size on the rate of flavor release depends on the nature of the system [8]. In the study conducted by Wendin, Langton, Caous and Hall [16], smaller droplet sizes in cream cheese resulted in a shorter duration of the dynamic sensation of “fat-creamy”.

The samples showed significant textural differences linked to the intensity of emulsification, with a more intense emulsification producing higher firmness and creaminess, as well as a decrease in adhesiveness to the spoon when handled. The textural attributes of mayonnaise can be explained by the elastic parameters of dynamic viscoelasticity (G’). This property is strongly related to particle size at 10% cumulative volume, which is in turn negatively correlated with sensory attributes including hardness, fracturability, and adhesiveness [13]. The perception of texture is a complex process involving the senses of vision, hearing, somesthesis, and kinesthesis [17]. Neurologically, texture perception results from the interaction of sensory and motor components of the peripheral nervous system with the central nervous system. Initially, the sight and extra-oral manipulation of food, e.g., through using a spoon, sets up sensory expectations regarding texture [18]. Then, once the food is put into the mouth, texture perception is a dynamic process, as the physical properties of foods change continuously when manipulated intra-orally. In this respect it would be interesting to further examine the question of how well texture attributes that are perceived extra-orally correlate with perceived oral-somatosensory attributes.

In the present study, the emulsion drop size distribution was not measured. However, previous investigations have shown that the scaling of drop-diameter averages with rotor tip-speed is highly predictable [9,10]. Using this previously established scaling, the higher emulsification intensity (rotor tip-speed 7.1 m/s compared to 4.7 m/s) corresponds to an expected reduction in the average oil drop diameter by a factor of two [9]. This is a rather substantial reduction that was expected to lead to quality differences with regard to the appearance, texture and flavor of the product. However, only extra-oral textural attributes were affected to a degree that could be perceived by the sensory panel. 

Our findings, that a more intense emulsification and hence a decreased oil drop diameter produces a firmer mayonnaise, compare well with earlier results regarding the effect of microstructure on food emulsions [13,16]. The instrumental texture analysis data support the theory that a decreased droplet size leads to textural alterations, resulting in a more firm and adhesive mayonnaise. These findings may be helpful for the control and prediction of mayonnaise texture using processing conditions rather than more common approaches such as adding texture modifiers, which in the age of growing consumer preference for “clean labels” are unwanted in many products. Understanding the microstructural changes that occur during processing and the role of different mayonnaise ingredients will allow for better control of product structure and, ultimately, the manipulation and regulation of product texture [17].

The study conducted by Maruyama, Sakashita, Hagura and Suzuki [13] is just one among many to report that temperature during preparation may affect the physical properties of mayonnaise. Thus, if the aim is to obtain reproducible results, a consistent temperature is essential. In the present study, the ingredients were left to adjust to room temperature at approximately 20 °C, with the mayonnaise thereafter prepared at the same temperature. Compared to industrial preconditions, in which emulsification is commonly performed under cooling, the temperature in this study was high and control was inadequate, which might have influenced the results. Since emulsion formation is controlled by viscous drop breakup [9], a high temperature at the onset of emulsification will decrease the viscosity of the emulsion, reducing the viscous shear forces and thus resulting in larger drop sizes. 

## 5. Conclusions

The effects of a higher emulsification intensity, corresponding to an expected reduction in the average oil droplet diameter by a factor of two, on the sensory and instrumental characteristics of full-fat mayonnaise were limited. Perceived and instrumentally measured texture was affected, with a more intense emulsification resulting in a firmer mayonnaise, as measured via back extrusion and by an analytical sensory panel. However, appearance, taste and flavor attributes were not affected by processing.

## Figures and Tables

**Figure 1:**
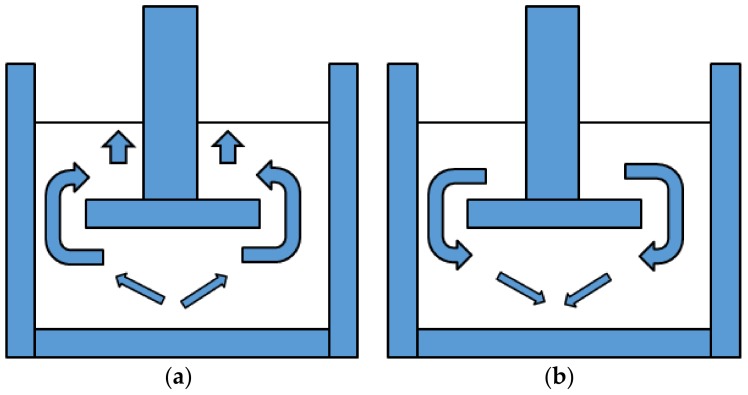
Movement of sample: (**a**) work of compression; (**b**) adhesiveness.

**Table 1:** List of ingredients in the experimental mayonnaise.

Ingredient	Weight (g)	*w*/*v* (%)
Rapeseed oil	321.6	81.2
Egg yolk	34.1	8.6
Water	23.3	5.9
Mustard	10	2.5
Vinegar (acetic acid 12%)	4.8	1.2
Salt	1.2	0.3
Sugar	1.2	0.3

**Table 2:** List of ingredients in the commercial reference sample.

Ingredient	*w*/*v* (%)
Rapeseed oil	81
Vinegar	
Egg yolk	4.6
Mustard seeds	
Sugar	
Salt	0.7
White pepper	
Thickener (E401, E412, E417)	
Cayenne pepper	
Preservatives(E211)	
Colorant, beta-carotene	

**Table 3:** Sensory attributes and definitions established by the panel.

Category	Attribute	Definition
Appearance	Shiny	Degree of shininess
	Yellow	Gradation from a weak to a strong tone of (vanilla) yellow
Texture (extra-oral)	Adhesiveness to spoon	Amount of mayonnaise remaining on the spoon when held vertically
	Firmness	Degree of resistance when stirring with a spoon
Texture (intra-oral)	Fatty mouthfeel	Graded from a little to a high grade of perceived fattiness
	Creaminess	Degree of creaminess; yoghurt used as reference
Taste	Acidity	Taste of sourness; vinegar and lemon used as reference
	Sweetness	The pure taste of sucrose; no reference used, evaluation relied on individual recollection of sweet taste
	Saltiness	The pure taste of sodium chloride; no reference used, evaluation relied on individual recollection of salty taste
Flavor	Egg flavor	Sulfur, boiled egg; boiled eggs used as reference
	Total flavor	The total intensity of taste and flavor

**Table 4:** Texture properties measured.

Texture Property	Definition
Firmness (g)	The maximum compression force
Work of compression (g·mm)	The ability of the sample to flow around the probe (see Figure 1)
Stickiness (g)	The maximum (negative) force recorded during the withdrawal phase.
Adhesiveness (g∙mm)	The work required to withdraw the probe through the sample (see Figure 1)
Gradient 1 (g/mm)	The gradient of the first third of the compression distance
Gradient 2 (g/mm)	The gradient of the second third of the compression distance

**Table 5:** Sensory evaluation of the intensity of selected attributes, comparing the experimental mayonnaises and the commercial reference. Different letters in the same row indicate significant differences at *p* ≤ 0.05.

Sensory Attribute	High Emulsification Intensity	Low Emulsification Intensity	Commercial Reference Mayonnaise
Shiny	62 ± 12 ^a^	65 ± 9 ^a^	70 ± 11 ^a^
Yellow	43 ± 11 ^a^	41 ± 12 ^a^	84 ± 8 ^b^
Adhesiveness to spoon	40 ± 17 ^a^	58 ± 19 ^b^	28 ± 14 ^c^
Firmness	54 ± 9 ^a^	47 ± 11 ^b^	70 ± 12 ^c^
Fatty mouthfeel	58 ± 17 ^a^	57 ± 14 ^a^	58 ± 14 ^a^
Creaminess	60 ± 9 ^a^	56 ± 13 ^a^	70 ± 11 ^b^
Acidity	34 ± 15 ^a^	33 ± 11 ^a^	55 ± 14 ^b^
Sweetness	29 ± 10 ^a^	28 ± 9 ^a^	24 ± 9 ^a^
Saltiness	23 ± 7 ^a^	23 ± 7 ^a^	28 ± 11 ^a^
Egg flavor	32 ± 10 ^a^	32 ± 9 ^a^	35 ± 7 ^a^
Total flavor	45 ± 10 ^a^	43 ± 9 ^a^	61 ± 13 ^b^

Different letters in the same row indicate significant differences at *p* ≤ 0.05.

**Table 6:** Instrumental texture analysis as performed via a back extrusion method. Different letters in the same row indicate significant differences at *p* ≤ 0.05.

	High Emulsification Intensity	Low Emulsification Intensity	Commercial Reference Mayonnaise
Firmness (g)	252 ± 14 ^a^	230 ± 15 ^a^	292 ± 11 ^b^
Stickiness (g)	−301 ± 22 ^a^	−269 ± 19 ^a^	−349 ± 23 ^b^
Adhesiveness (J)	40 ± 3 ^a^	35 ± 5 ^a^	48 ± 3 ^b^
Gradient 1 (g/mm)	29 ± 1 ^b^	27 ± 1 ^a^	37 ± 11 ^a,b^
Gradient 2 (g/mm)	5 ± 2 ^a^	5 ± 1 ^a^	10 ± 3 ^a^

Different letters in the same row indicate significant differences at *p* ≤ 0.05.
